# Association of 410L, 1016I and 1534C *kdr* mutations with pyrethroid resistance in *Aedes aegypti* from Ouagadougou, Burkina Faso, and development of a one-step multiplex PCR method for the simultaneous detection of 1534C and 1016I *kdr* mutations

**DOI:** 10.1186/s13071-023-05743-y

**Published:** 2023-04-19

**Authors:** Aboubacar Sombié, Wendegoudi Mathias Ouédraogo, Manabu Oté, Erisha Saiki, Tatsuya Sakurai, Félix Yaméogo, Antoine Sanon, Philip J. McCall, Hirotaka Kanuka, David Weetman, Athanase Badolo

**Affiliations:** 1 Laboratoire d’Entomologie Fondamentale et Appliquée, Université Joseph Ki-Zerbo, Ouagadougou, Burkina Faso; 2 Programme National de Lutte Contre Les Maladies Tropicales Négligées, Ministère de la Santé, Ouagadougou, Burkina Faso; 3 Center for Medical Entomology, The Jikei University School of Medicine, Tokyo, Japan; 4 Department of Tropical Medicine, The Jikei University School of Medicine, Tokyo, Japan; 5 Laboratory Animal Facilities, The Jikei University School of Medicine, Tokyo, Japan; 6 Department of Vector Biology, Liverpool School of Tropical Medicine, Liverpool, UK

**Keywords:** *Aedes aegypti*, Pyrethroids, Resistance, Malathion, Multiplex PCR, *Kdr*, Burkina Faso, Dengue

## Abstract

**Background:**

Since 2000, Burkina Faso has experienced regular dengue cases and outbreaks, making dengue an increasingly important health concern for the country. Previous studies in Burkina Faso reported that resistance of *Aedes aegypti* to pyrethroid insecticides was associated with the F1534C and V1016I *kdr* mutations. The current study reports high resistance of *Ae. aegypti* populations to pyrethroid insecticides, likely supported by mutations in the voltage-gated sodium channel, here evidenced by genotyping the *kdr* SNPs V410L, V1016I and F1534C. We also describe a new multiplex PCR-based diagnostic of F1534C and V1016I *kdr* SNPs.

**Methods:**

Larvae of *Ae. aegypti* were collected from three health districts of Ouagadougou in 2018. The resistance status of *Ae. aegypti* to permethrin (15 μg/ml) and deltamethrin (10 μg/ml) was tested using bottles and to malathion (5%) using WHO tube tests. All bioassays used 1-h exposure and mortality recorded 24 h post-exposure. Bioassay results were interpreted according to WHO thresholds for resistance diagnosis. The *kdr* mutations were screened using AS-PCR and TaqMan methods in exposed and non-exposed *Aedes* mosquitoes.

**Results:**

Females from all health districts were resistant to permethrin and deltamethrin (< 20% mortality) but were fully susceptible to 5% malathion. The F1534C and V1016I *kdr* mutations were successfully detected using a newly developed multiplex PCR in perfect agreement with TaqMan method. The 1534C/1016I/410L haplotype was correlated with permethrin resistance but not with deltamethrin resistance; however, the test power was limited by a low frequency of dead individuals in deltamethrin exposure.

**Conclusions:**

Resistance to pyrethroid insecticides is associated with *kdr* mutant haplotypes, while the absence of substantial resistance to malathion suggests that it remains a viable option for dengue vector control in Ouagadougou.

**Supplementary Information:**

The online version contains supplementary material available at 10.1186/s13071-023-05743-y.

## Background

*Aedes aegypti* is a prolific mosquito with worldwide distribution, transmitting important arboviruses such as dengue, yellow fever, chikungunya and Zika. Dengue is by far the most important arbovirus with 390 million cases and 25,000 deaths recorded annually worldwide [1]. The most important contemporary dengue outbreak in Burkina Faso occurred in 2017 with 14,455 recorded cases and 29 deaths [2] and involved three (DENV-1, DENV-2 and DENV-3) of the four dengue virus serotypes [3]. With increasing urbanization and associated human population density, the West African region is particularly at risk of dengue [4]. *Aedes aegypti* is closely associated with urban environments, and in West Africa, it breeds preferentially in human-made breeding sites such as plastic containers, drums, used tires and miscellaneous medium and small containers [5]. Insecticide-based vector control and breeding site reduction are the only effective dengue prevention methods [6] available in the absence of an effective dengue vaccine which can protect against all dengue virus serotypes [7]. However, the intensive use of insecticides has led to development worldwide of resistance in *Ae. aegypti* populations [8] driven primarily by target site mutations and metabolic resistance mechanisms [8, 9].

Multiple target site knockdown resistance (*kdr*) mutations that affect pyrethroid susceptibility have been identified in *Ae. aegypti* of which the most common is the F1534C mutation. This mutation has a worldwide distribution [8] and is frequently found associated with the V1016G and S989P *kdr* mutations in Asia and the V1016I *kdr* mutation in Latin America [10] and Africa [11, 12]. In addition, another important variant, V410L (V419L using *Ae. aegypti* codon numbering), in the first domain of the voltage-gated sodium channel (*Vgsc*), has been detected more recently [13, 14]. Originally reported in a laboratory strain in Brazil [15], subsequent reports have shown high frequencies of this mutation in Colombia [16] and Mexico, with steep increases seen since it was first detected over 15 years ago [13, 17]. Most recently V410L has been reported in Central and West Africa [18, 19]. Several of the *Vgsc* mutations, including V1016I, are not associated with pyrethroid resistance when they occur alone [20], while V410L, V1016G and F1534C can confer resistance but with effects amplified by co-presence of additional mutants [21].

The current study reports resistance to pyrethroid insecticides at least partially underpinned by a trio of *kdr* mutations. Also, a new PCR diagnostic method is proposed for the simultaneous detection of the 1534C and 1016I *kdr* mutations. Lack of substantial resistance to malathion suggests continued utility as an approved alternative for control of *Aedes aegypti* in Ouagadougou.

## Methods

### *Aedes aegypti* larval collection and mosquito rearing

*Aedes aegypti* immature stages (larvae and pupae) were collected in Ouagadougou in 2018 from three health districts (Baskuy, Bogodogo and Nongremassom). These health districts were selected based on variable dengue burden reported during the 2016 outbreak, with Bogodogo and Nongremassom having recorded the highest numbers of dengue cases while Baskuy recorded few cases. Immature stage mosquitoes were collected from tires in Bogodogo and Nongremassom and from plastic containers in Baskuy. Signed informed consent was required from house owners when collecting from breeding containers situated inside households. Collections were transported to the insectary of Université Joseph Ki-Zerbo at Ouagadougou and reared to adults using Tetramin®. Emerged adults were provided with 10% sugar solution. All life stages were maintained at 25–27 °C temperature and 75–85% relative humidity.

### Insecticide bioassays

Bioassays with pyrethroid insecticides were performed using 250-ml Wheaton bottles with adaptation of the CDC bioassay protocol [22] using 1 h exposure and 24 h delay mortality record. Malathion-impregnated papers were ordered from WHO reference center in Malaysia and technical grade pyrethroid insecticides were order from Sigma Aldrich. A batch of 25 unfed female adults of 3–5-day-old *Ae. aegypti* mosquitoes was exposed for 1 h to insecticide in four bottles coated at the recommended concentrations: 15 μg/bottle for permethrin and 10 μg/bottle for deltamethrin dissolved in acetone. An additional bottle coated with acetone only was used as negative control. Bioassays with malathion (5%) were carried out using the WHO tube bioassay with approximately 25 mosquitoes per tube or bottle, for a total of 100 mosquitoes per insecticide and per population [23]. All bioassays were carried out once per insecticide and per population, in parallel with susceptible Rockefeller laboratory strain (eggs kindly donated by the Powell Labs, Department of Ecology and Evolutionary Biology, Yale University), at 27 °C with 82 ± 3% relative humidity. The number of knocked down mosquitoes was counted every 10 min for a total period of 1 h. After 1 h exposure, mosquitoes were transferred to either cardboard cups covered in mesh following the bottle bioassay or holding tubes following the tube bioassay. The number of dead mosquitoes was recorded at 24 h after bioassay. Mortality rates were calculated and adjusted with Abbott’s correction [24] when the mortality in the control tube was between 5 and 20% for both tube and bottle bioassays. Dead and alive mosquitoes were stored separately in 1.5-ml tubes over silica gel for molecular analysis.

### DNA extraction

DNA was extracted from 431 mosquitoes including 407 from pyrethroid-coated bottles and 24 from control bottles. The control mosquitoes were used to develop the multiplex method, and those exposed to pyrethroid were used to test the association of resistance phenotypes and genotypes. For multiplex and AS-PCR, mosquito DNA was extracted following a previously described protocol [12], and the DNA pellet was suspended in 20 μl of TE buffer and stored in a freezer until use while for TaqMan method extraction was performed as described in Badolo et al. [25].

### Multiplex PCR detection of the F1534C and V1016I *kdr* mutations

To simplify genotyping of two common *kdr* mutants, a multiplex PCR was developed using known genotypes from eight previously sequenced *Ae. aegypti* DNAs [12] as positive samples. For large detection to compare with AS-PCR method, 431 mosquitoes in total were used. F1534C detection primers [26] and V1016I detection primers [27] were mixed in a single tube for the detection of the two mutations (Additional file 1: Table S1). In total, seven primers were mixed in the single PCR reaction tube to simultaneously characterize the two SNPs (F1534C and V1016I).

**Fig. 1:**
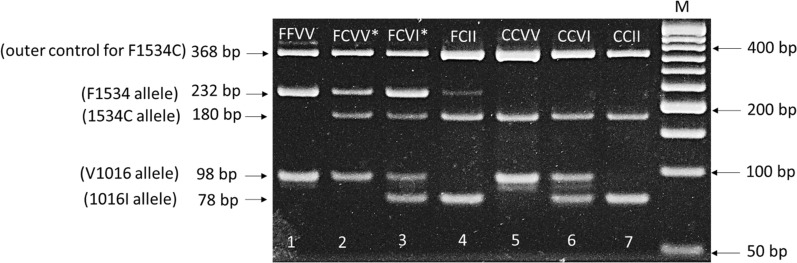
Electrophoresis gel showing genotypes of double-mutation *kdr* F1534C/V1016I. FFVV (1534FF/1016VV), FCVV (1534FC/1016VV), FCVI (1534FC/1016VI), FCII (1534FC/1016II), CCVV (1534CC/1016VV), CCVI (1534CC/1016VI), CCII (1534CC/1016II). M, ladder marker. *Genotypes not detected in our field *Aedes* populations. Lane 1: DNA sample from *Aedes* susceptible strain Rockefeller, lane 2–3: DNA from known sequenced genotypes, lane 4–7: DNA sample from field *Aedes* of the study area

The PCR reaction contained 1 μl target DNA, 12.5 μl DNA polymerase mix, (AmpliTaq, Thermo Fisher Scientific), 0.5 μl c1534-f and c1534-r primers (1 μM), 0.125 μl Ae1534F-r, Ae1534C-f, Iso1016f and Iso1016r primers (0.25 μM) and 0.25 μl Val1016f primer (0.5 μM). The PCR reaction volume was completed to 25 μl with distilled water. The PCR was performed on a T100™ Thermal Cycler (Bio-Rad Laboratories, Inc.) with the following program: 95 °C/5 min for 1 cycle, (95 °C/30 s, 55 °C/30 s, 72 °C/45 s) for 30 cycles and 72 °C/10 min for 1 cycle, maintained at 4 °C after the PCR was completed. PCR products were mixed with 5 μl of 6 × loading buffer and loaded on 3% TBE agarose gel. After 1 h of electrophoresis in 0.5% TBE buffer, the gel was stained for 10 min in 0.5 mg/ml ethidium bromide and de-stained in distilled water for 5 min for visualization of bands under UV light (Fig. 1).

### V410L* kdr* mutation genotyping by allele-specific (AS) PCR

A total of 431 dead and alive mosquitoes from permethrin and deltamethrin exposure were used for AS-PCR detection of the *kdr* mutations. The V410L *kdr* mutation was screened using AS-PCR described by Granada et al. [16]. The wild-type and mutant alleles were detected in two different PCR reactions. Briefly, each PCR reaction contained 1 μl of target DNA, 6.25 μl of DNA polymerase mix (AmpliTaq, ThermoFisher Scientific) and 0.3 μM of each primer (Additional file 1: Table S1). The PCR reaction volume was completed to 12.5 μl with distilled water. The PCR conditions were 95 °C for 5 min and 35 cycles of 95 °C for 30 s, 60 °C for 30 s and 72 °C for 1 min, followed by a final extension step of 72 °C for 7 min. PCR products were mixed with 6 × loading buffer, and 5 μl of the mixture was loaded on a 1.5% TAE agarose gel. After electrophoresis and ethidium bromide staining, band sizes were interpreted according to Granada et al. [16].

### TaqMan detection of *kdr* mutations

A total of 134 mosquitoes were screened by a singleplex TaqMan qPCR for the cross-validation of the V1016I and F1534C mutants detected in the novel multiplex assay and V410L *Vgsc* mutations [28]. Briefly, reactions were performed in 96-well plates by adding 5 μl TaqMan gene expression SensiMix (Applied Biosystem, Foster City, CA, USA), 0.125 μl primer/probe, 3.875 μl molecular grade sterile water and 1 μl DNA extract [25]. Reactions were run on an Agilent MX3000P qPCR thermal cycler using cycling conditions of an initial denaturation of 10 min at 95 °C, followed by 40 cycles of 92 °C for 15 min and 60 °C for 1 min.

### DNA sequencing for confirmation of the V410L mutation

DNA sequencing was used as a gold standard to validate the genotyping of the V410L *kdr* mutation. A partial sequence of domain I of the voltage-gated sodium channel of eight samples was amplified by PCR in a 25-μl volume with iCycle thermal cycler (Bio-Rad Laboratories). Each reaction contained 12.5 μl PrimeSTAR^®^ Max DNA polymerase (Takara Bio Inc.), 1.5 μl (0.3 μM) reverse primer described by Granada et al. [16] and a newly designed forward primer to increase the amplification efficiency (Additional file 1: Table S1); sterile water was added to make a final volume of 25 μl. Thermal cycling conditions were one cycle of denaturation at 98 °C for 5 min, followed by 35 cycles of 98 °C/10 s, 60 °C/15 s and 72 °C/5 s, ending with one cycle at 72 °C for 7 min for the final extension. Ten microliters of the mixture of PCR products and 6 × loading buffer were run on a 1.5% TAE agarose gel, and the expected DNA band size was read at 688 bp. The DNA fragment was cut from the gel and purified from gel using a QIAEX^®^ II Gel Extraction Kit according to manufacturer's instructions. Purified PCR products were sequenced by Fasmac sequencing service (FASMAC Co., Ltd., Japan). Sequence alignment was performed with Snap Gene^®^ Viewer 5.0.5 software.

### Data analysis

Insecticide bioassay data were interpreted according to WHO guidelines based on after 24 h delayed mortality: mosquitoes were considered susceptible if the mortality rates were > 98%, possibly resistant if mortality rates were between 90 and 97% and resistant if mortality rates were < 90% [29, 30]. All statistical analyses were performed using R version 3.4.3 with Stats and Genetics packages [31].

Chi-square and Kruskal-Wallis tests were used to compare *kdr* allele, genotype and haplotype frequencies between insecticide phenotypes (live or dead) and among *Aedes* larval collection localities.

Significance of the spatial variations in mortality of *Ae. aegypti* adults due to pyrethroid insecticide (permethrin and deltamethrin) was evaluated using a generalized linear model (GLM) with binomial link function under R Stats package version 4.0.5. Linkage disequilibrium between the two *kdr* mutations, V410L and V1016I, was quantified (as r^2^) using R Genetics package version 1.3.8.1.3. Data were pooled across the three districts to increase sample size to detect linkage disequilibrium and to test phenotype-*kdr* genotype associations.

## Results

### Susceptibility status of *Aedes aegypti* to pyrethroid and malathion insecticides

**Fig. 2:**
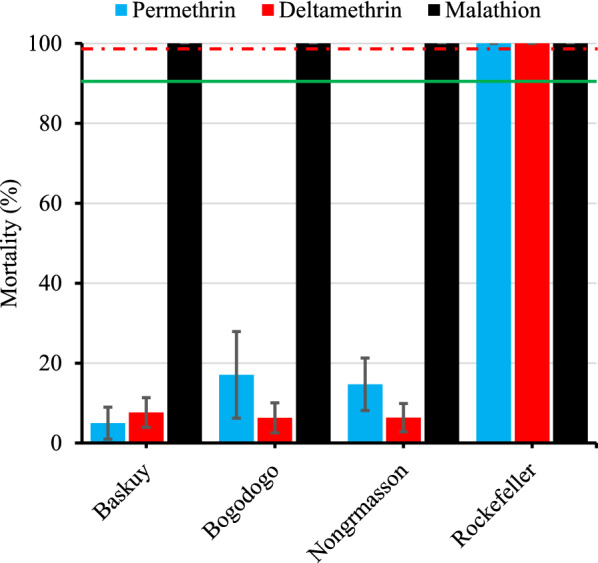
*Aedes aegypti* mortality rate (%) after 24-h bottle bioassays with pyrethroids and WHO tube bioassay with malathion. The green line indicates the resistance threshold while the red dashed line indicates the susceptibility threshold. The 95% confidence bars are also indicated

The *Ae. aegypti* populations in all three health districts were found to be resistant to permethrin and deltamethrin at the tested concentrations, with very low mortality rates (< 20%) (Fig. 2). As expected, the Rockefeller strain was fully susceptible to both insecticides (100% mortality). A similar trend was observed when considering knockdown time for 30 min (KDT30) (Additional File 2: Figure S1). While overall mortality did not differ significantly between insecticides or localities, the significant insecticide × locality interaction term in the generalized linear model indicates some inconsistency. In particular, permethrin mortality in Bogodogo and Nongremassom was significantly higher than deltamethrin mortality in Baskuy (Additional file 1: Table S2).

### Allele and genotype frequencies of V410L, V1016I and F1534C *kdr* mutations using multiplex and TaqMan

Of the 134 samples screened by the TaqMan method for the F1534C and V1016I *kdr*, 27 (20.15%), 59 (44.03%) and 47 (35.07%) were identified as CCVV, CCVI and CCII genotypes, respectively. The multiplex PCR method returned the same genotypes in 133 of the samples. Only one sample (0.75%) could not be successfully amplified with the TaqMan method for the 1534 SNP, but was scored as 1534 CC with the newly developed multiplex method.

**Fig. 3:**
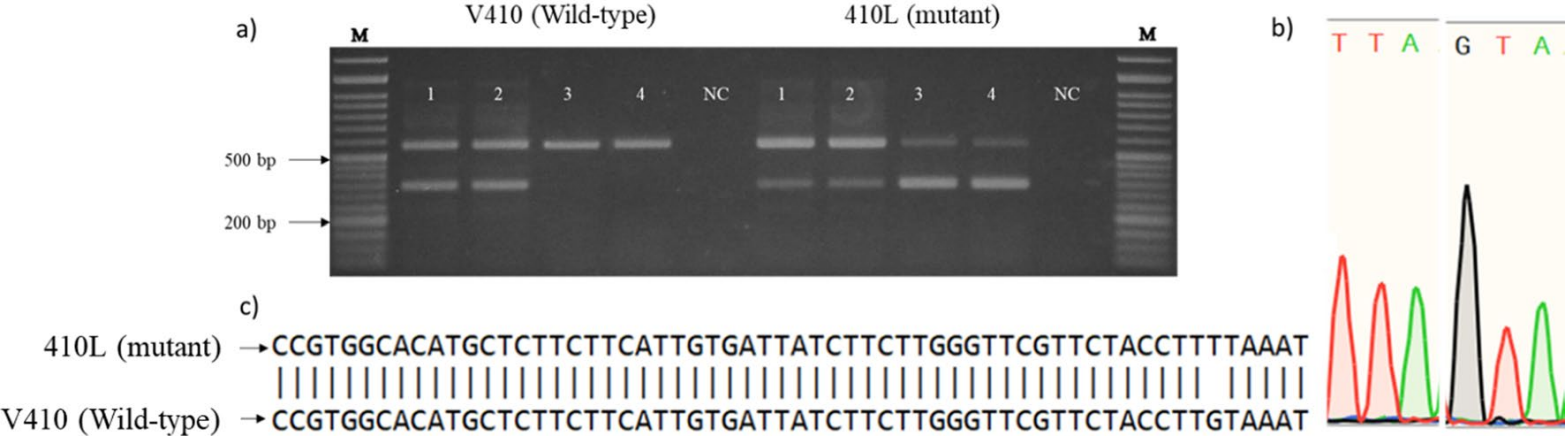
Wild-type and *kdr* allele detection of V410L mutation. **a** Electrophoresis gel showing genotypes of V410L mutation. M, ladder marker. Lane 1–4: DNA sample, NC: negative control, **b** chromatogram of mutant (TTA) and wild-type (GTA) alleles of V410L *kdr* mutation, **c** DNA sequence of mutant and wild-type alleles of V410L *kdr* mutation

Detection of the V410L *kdr* mutation in *Ae. aegypti* was confirmed by TaqMan in the same 134 mosquito samples with total agreement. Additionally, direct DNA sequencing in eight *Ae. aegypti* mosquitoes confirmed the expected substitution of G by T (GTA → TTA) (Fig. 3).

### V410L, V1016I and F1534C *kdr* frequencies and triple-locus genotype associations with insecticide resistance

**Fig. 4:**
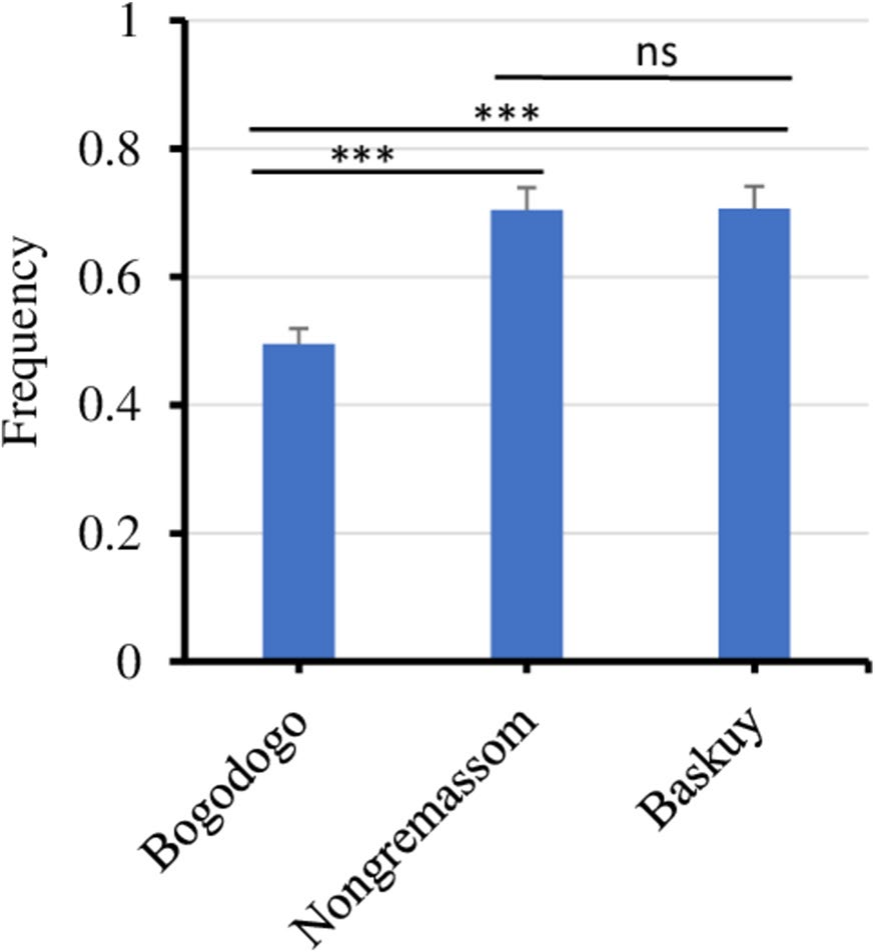
Variation of 1016I/410L kdr allele frequencies of Aedes aegypti populations between health districts. ***Significantly different (P<0.001),  ns: non-significant. 1534C kdr allele was not considered in this graph because of it almost fixation in all collection sites

Genotyping of F1534C, V1016I and V410L *kdr* mutations was carried out on 431 *Ae. aegypti* mosquitoes in total, which included 407 pyrethroid-exposed and 24 control mosquitoes (Additional file 1: Table S3). The frequencies of the 410L, 1016I and 1534C *kdr* alleles (calculated based on the 431 samples) were similar in Baskuy and Nongremassom but significantly lower in Bogodogo (Fig. 4). The V1016I and V410L *kdr* mutations were in near perfect linkage disequilibrium (*r*^2^ = 0.977). Of the 27 possible genotypes, five were recorded in the three localities, with CIL/CIL, CVV/CIL and CVV/CVV being the most representative ones (Table 1). The two other genotypes, CIL/CIV and CIL/FIL, were rare, and 22 were absent in the populations of *Ae. aegypti*. No statistical difference was observed among the three localities regarding the distribution of genotypes.

**Table 1:** Triple-locus *kdr* genotype and haplotype of F1534C, V1016I and V410L mutations and their frequencies

Locality	Genotype frequency					Haplotype frequency			
	CIL/CIL	CIL/CIV	CIL/CVV	CVV/CVV	CIL/FIL	CIL	CIV	CVV	FIL
Baskuy	0.500	0.028	0.366	0.106	0.000	0.69	0.016	0.295	0
Bogodogo	0.247	0.019	0.468	0.266	0.000	0.486	0.017	0.497	0
Nongremassom	0.474	0.015	0.421	0.083	0.008	0.683	0.018	0.295	0.004

C, I and L are the mutant alleles of F1534C, V1016I and V410L while F, V and V are the wild-type alleles for these *kdr* mutations, respectively

Only four haplotypes were recorded across the three *kdr* mutations with the CIL haplotype (1534C/1016I/410L) being the most common. CIL was found at the highest frequency of 48.6%, 68.3% and 69.0%, respectively, in Bogodogo, Nongremassom and Baskuy, with non-significant difference between the collection sites (χ^2^ = 0.61, *df* = 2, *P* = 0.738) (Table 1).

On the other hand, CIL was found significantly associated with permethrin resistance (χ^2^ = 36.37,  *df* = 1, *P* < 0.001) with more than tenfold resistance advantage (3.7% mortality) compared to the single mutant CVV haplotype (42.9% mortality). In contrast, for deltamethrin resistance, no association of CIL haplotype to resistance was recorded (χ^2^ = 0.031, *df* = 1, *P* = 0.861), though the very low number of dead individuals limited test power (Table 2).

**Table 2:** Triple-locus *kdr* genotypes of F1534C, V1016I and V410L mutations in dead and alive *Ae. aegypti* mosquitoes and the association of their haplotypes with pyrethroid resistance

Genotypes	Alive	Dead	Mortality (%) (95% CI)	CIL vs. CVV
Permethrin				*χ*^*2*^*; df, p*-value
CIL/CIL	104	4	3.7 (1.45–9.14)	*χ*^*2*^ = 36.37, *df* = 1, *P* < 0.001
CVV/CIL	97	16	14.16 (8.91–21.77)	
CVV/CVV	24	18	42.86 (29.12–57.79)	
Deltamethrin				
CIL/CIL	60	3	4.76 (1.63–13.09)	*χ*^*2*^ = 0.031, *df* = 1, *P* = 0.861
CVV/CIL	52	4	7.14 (2.81–16.98)	
CVV/CVV	21	1	4.55 (0.81–21.80)	

C, I and L are the mutant alleles of F1534C, V1016I and V410L while F, V and V are the wild-type alleles for these *kdr* mutations respectively

## Discussion

Here, we developed a multiplex PCR for simultaneous detection of F1534C and V1016I *kdr* mutations in one reaction tube, useful to save time and resources, compared to detecting each mutation individually. The results are comparable to those from an established method to detect the mutants separately, with complete agreement where full genotypes were available from each method. *Aedes aegypti* populations from Baskuy, Bogodogo and Nongremassom health districts were susceptible to malathion at the dose tested, as previously reported in some localities of Burkina Faso including elsewhere in Ouagadougou [25, 32]. Noting that the malathion dose used in the study was > threefold higher than the diagnostic dose of 1.5% [30] for *Aedes* mosquitoes, the test result indicates a lack of substantial resistance rather than full susceptibility. These results reflect those from a previous review of *Ae. aegypti* malathion resistance across Africa [4] and more recent studies from Senegal with susceptibility to diagnostic doses of 0.8% and 5% [33] and to 5% malathion in Sudan [34] and Côte d’Ivoire [35]. This suggests that malathion remains a viable option for control of *Ae. aegypti* populations during dengue outbreaks in Africa, in contrast with at least some populations from America [36] and Asia [37].

*Aedes aegypti* populations were resistant to deltamethrin and permethrin confirming previous reports from elsewhere in Ouagadougou [12, 25]. This resistance is consistent with a role of *kdr* mutations, with near fixation of the 1534C mutant and high frequency of the 1016I and 410L alleles. The F1534C *kdr* mutation was found at very high frequency (99.8%) in Ouagadougou, in agreement with previous frequency of 0.97 recorded 2 years before [25] and 0.92 1 year later [19], demonstrating the stability and the fixation of this allele in Ouagadougou, with comparable results recorded in Ghana recently [38]. The 1016I *kdr* allelic frequency has increased rapidly in Ouagadougou within a 2-year period (χ^2^ = 97.22, *df* = 1, *P* < 0.0001) from 0.20 and 0.47 in previous collections in 2016 [12, 25] to 0.64 in the current study (2018). Malaria vector control including upscaling of ITN distribution (effect on resting *Ae. aegypti*, not blood-feeding [39]) in West Africa and individual use of insecticides (aerosols, coils) [40] may have contributed to the selection of *Ae. aegypti* resistance to pyrethroid insecticides and increased *kdr* allele frequencies [41]. The V410L *kdr* mutation was found at high frequency (0.62) and in near-perfect linkage disequilibrium with V1016I suggesting that this mutation has been present but undetected in Burkina Faso for some time. Toé et al. [19] detected V410L *kdr* mutation in Burkina Faso at frequencies ranging from 0.1 in Banfora to 0.36 in Ouagadougou using a real-time melting curve qPCR method. The 410L allele frequency reported for Ouagadougou is lower than that found in the current study, suggesting possible spatial and/or temporal fluctuation.

The V1016I, V410L and F1534C *kdr* alleles co-occur in the *Ae. aegypti* population from Burkina Faso. The lack of F1534C, V1016I and V410L genotype and haplotype diversity is linked to the fixation of the 1534C allele in Ouagadougou as reported in previous studies. The situation is different from other parts of the world where diversity of haplotypes and genotypes is recorded, suggesting that these mutations do not occur independently. In Madeira [13] and Luanda [18], respectively, there are 6 and 12 genotypes, while in the Americas, more diversity is recorded with 23 genotypes in Harris County (USA) [42], 20 genotypes in Mexico [13] and 14 haplotypes in Columbia [16]. The F1534C, V1016I and V410L *kdr* mutations may have displayed similar sequential dynamics as in Colombia [16] and Mexico [13] with positive selection for the first occurring 1534C mutation followed by association of the second and third mutations. While we found significant positive association of the 1534C/1016I/410L haplotype with permethrin resistance, lack of a significant association with deltamethrin resistance does not necessarily mean lack of any contribution of the *kdr* haplotype to phenotype, but rather, may reflect low power from the very low number of dead individuals. However, a contribution from other resistance mechanisms, perhaps including P450 enzyme overexpression [25], cannot be ruled out for either pyrethroid phenotype. The driver(s) of the increase in *kdr* genotype frequencies are unclear but recent studies in Ouagadougou recorded that 50% of households use pyrethroid insecticides as sprays or coils, and pyrethroid-treated ITNs were found in approximately 80% of households [5]. Such pressures could readily lead to the selection for *Ae. aegypti* carrying *kdr* alleles. The spatial and temporal intensification of pyrethroid resistance and the more likely evolution to the fixation of 410L and 1016I *kdr* mutations recommend actions for surveillance and alternatives to pyrethroid-based methods of control [43]. With recent successes in *Wolbachia*-infected *Ae. aegypti* mosquito releasing for dengue control [44] and progress in CRISPR/*Cas9*-based gene drive development for *Ae. aegypti* [45], deployment of alternative methods can be expected in the coming years.

## Conclusion

*Aedes aegypti* collected from three health districts in Ouagadougou, Burkina Faso, were strongly resistant to deltamethrin and permethrin. Permethrin resistance was associated with *kdr* mutations, including the recently detected 410L mutant, which was recorded at high frequencies. A multiplex PCR was developed and validated to detect simultaneously the F1534C and V1016I *kdr* mutations to complement the AS-PCR available for 410L for insecticide resistance surveillance.

## Supplementary Information

**Additional file 1**: **Table S1**. List of primers sequences used for detecting V410L, F1534C and V1016I *kdr* mutations of *Aedes aegypti* from Ouagadougou health district. **Table S2**. Generalized linear model of *Ae. aegypti* mortality rates between insecticides and localities. Reference factor levels of predictors are shown in brackets, with beta effect size estimates, confidence intervals, z-value and associated probabilities for predictors included in the model. Significant predictor terms are shown in bold. **Table S3**. Genotype number and *kdr* allele frequencies of V410L, V1016I and F1534C *kdr* mutations in *Ae. aegypti *mosquito samples from Baskuy, Bogodogo and Nongremassom health district of Ouagadougou.

**Additional file 2**: **Figure. S1**. *Aedes aegypti *knockdown time 30 min rate (%) with CDC bottle pyrethroid bioassays. The black line indicates the resistance threshold while the red dashed line indicates the susceptibility threshold; 95% CI bars are also indicated.

## Data Availability

The data of the current study are available from the corresponding author on request.
