# Supplementary material for: Association of 410L, 1016I and 1534C kdr mutations with pyrethroid resistance in Aedes aegypti from Ouagadougou, Burkina Faso, and development of a one-step multiplex PCR method for the simultaneous detection of 1534C and 1016I kdr mutations
Source: Parasit Vectors. 2023 Apr 19;16:137. doi: 10.1186/s13071-023-05743-y (PMC10116651; doi:10.1186/s13071-023-05743-y)
Supplement: Supplementary file 1 — Additional file 1: Table S1. List of primers sequences used for detecting V410L, F1534C and V1016I kdr mutations of Aedes aegypti from Ouagadougou health district. Table S2. Generalized linear model of Ae. aegypti mortality rates between insecticides and localities. Reference factor levels of predictors are shown in brackets, with beta effect size estimates, confidence intervals, z-value and associated probabilities for predictors included in the model. Significant predictor terms are shown in bold. Table S3. Genotype number and kdr allele frequencies of V410L, V1016I and F1534C kdr mutations in Ae. aegypti mosquito samples from Baskuy, Bogodogo and Nongremassom health district of Ouagadougou. [file 13071_2023_5743_MOESM1_ESM.docx]

**Additional file 1: Table S1:** List of primers sequences used for detecting V410L, F1534C and V1016I kdr mutations of *Aedes aegypti* from Ouagadougou health district

| Kdr mutations | Primers sequences | References |
| --- | --- | --- |
| V410L | *kdr genotyping*  PM1_Ext_419F: GAT TCC TCC AGA ACT CCA CC  PM1_Ext_419R: TCA ATG GAT TTG GGT GAC AA  PM1_F_419Wt: CTT GGG TTC GTT CTA CCT TG  PM1_F_419Mut: CTT GGG TTC GTT CTA CCT TT  *Direct sequencing*  PM1_Ext_419R: TCA ATG GAT TTG GGT GAC AA  BF113F: CGC CAA CGT GAA ATA CCA TG | [16]  [16]  This study |
| F1534C | *kdr genotyping*  c1534-f: GCG TAC CTG TGT CTG TTC CA  c1534-r: GGC TTC TTC GAG CCC ATC TT  Ae1534F-r: GCG TGA AGA ACG ACC CGA  Ae1534C-f: CCT CTA CTT TGT GTT CTT CAT CAT CTG | [26] |
| V1016I | *Kdr genotyping*  Val1016f: GCG GGC AGG GCG GCG GGG GCG GGG CCA CAA ATT GTT TCC CAC CCG CAC CGG  Iso1016f: GCG GGC ACA AAT TGT TTC CCA CCC GCA CTG A  Iso1016r: GGA TGA ACC GAA ATT GGA CAA AAG C | [27] |

**Additional file 1: Table S2:** Generalised linear model of *Ae. aegypti* mortality rates between insecticides and localities. Reference factor levels of predictors are shown in brackets, with beta effect size estimates, confidence intervals, z-value and associated probabilities for predictors included in the model. Significant predictor terms are shown in bold.

| **Predictors** | Estimate | 95%CL | z-value | Pr(>\|z\|) |
| --- | --- | --- | --- | --- |
| **Intercept** | **-2.47** | **[-3.00** - **1.94]** | **-9.18** | **<0.001** |
| Insecticide [Deltamethrin] |  |  |  |  |
| Permethrin | -0.54 | [-1.39 - 0.31] | -1.24 | 0.22 |
| Locality [Baskuy] |  |  |  |  |
| Bogodogo | -0.22 | [-0.99 - 0.55] | -0.56 | 0.58 |
| Nongremassom | -0.26 | [-1.07 - 0.54] | -0.64 | 0.52 |
| Insecticide [Deltamethrin]: Locality [Baskuy] |  |  |  |  |
| **Permethrin: Bogodogo** | **1.68** | **[0.60 - 2.77]** | **3.04** | **<0.01** |
| **Permethrin: Nongremassom** | **1.49** | **[0.36 - 2.62]** | **2.58** | **0.01** |

**Additional file 1: Table S3:** Genotype number and *kdr* allele frequencies of V410L, V1016I and F1534C *kdr* mutations in *Ae. aegypti* mosquito samples from Baskuy, Bogodogo, and Nongremassom health district of Ouagadougou.

| Locality | V410L genotype | | | Freq. of L allele | V1016I genotype | | | Freq. of I allele | F1534C genotype | | | Freq. of C allele |
| --- | --- | --- | --- | --- | --- | --- | --- | --- | --- | --- | --- | --- |
|  | LL | VL | VV |  | II | VI | VV |  | CC | FC | FF |  |
| Baskuy | 72 | 56 | 15 | 0.699 | 76 | 52 | 15 | 0.713 | 142 | 0 | 0 | 1.000 |
| Bogodogo | 38 | 75 | 41 | 0.490 | 41 | 72 | 41 | 0.500 | 154 | 0 | 0 | 1.000 |
| Nongremassom | 64 | 58 | 11 | 0.699 | 67 | 56 | 11 | 0.709 | 133 | 1 | 0 | 0.996 |
| Total | 174 | 189 | 67 | 0.624 | 184 | 180 | 67 | 0.636 | 429 | 1 | 0 | 0.999 |

C, I and L are the mutant alleles of F1534C, V1016I and V410L while F, V and V are the wild-type alleles for these kdr mutations respectively

**Additional file 2: Fig. S1:** *Aedes aegypti* Knockdown Time 30 min rate (%) with CDC-bottle pyrethroids bioassays**.** The black line indicates the resistance threshold while the red dashed line indicates the susceptibility threshold. CI 95% bars are also indicated

**
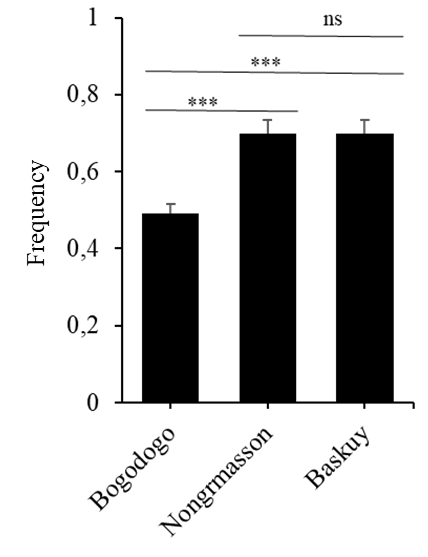
**

**Additional file 2: Fig. S2:** Variation of 1016I/410L kdr allele frequencies of *Ae.* *aegypti* populations between health districts. ***: significantly different (*P < 0.001*), ns: non-significant. 1534C kdr allele was not taking account in this graph due to it almost fixation in all collection sites.
