# Supplementary material for: Association of 410L, 1016I and 1534C kdr mutations with pyrethroid resistance in Aedes aegypti from Ouagadougou, Burkina Faso, and development of a one-step multiplex PCR method for the simultaneous detection of 1534C and 1016I kdr mutations
Source: Parasit Vectors. 2023 Apr 19;16:137. doi: 10.1186/s13071-023-05743-y (PMC10116651; doi:10.1186/s13071-023-05743-y)
Supplement: Supplementary file 2 — Additional file 2: Figure. S1. Aedes aegypti knockdown time 30 min rate (%) with CDC bottle pyrethroid bioassays. The black line indicates the resistance threshold while the red dashed line indicates the susceptibility threshold; 95% CI bars are also indicated. [file 13071_2023_5743_MOESM2_ESM.docx]

**Additional file 2: Fig. S1:** *Aedes aegypti* Knockdown Time 30 min rate (%) with CDC-bottle pyrethroids bioassays**.** The black line indicates the resistance threshold while the red dashed line indicates the susceptibility threshold. CI 95% bars are also indicated

**
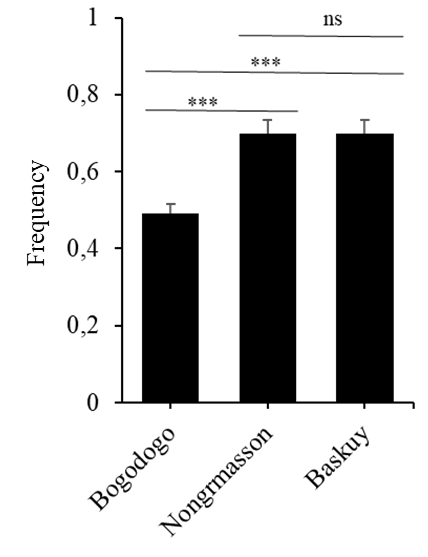
**

**Additional file 2: Fig. S2:** Variation of 1016I/410L kdr allele frequencies of *Ae.* *aegypti* populations between health districts. ***: significantly different (*P < 0.001*), ns: non-significant. 1534C kdr allele was not taking account in this graph due to it almost fixation in all collection sites.
